# Exogenous hormone therapy and non-melanoma skin cancer (keratinocyte carcinoma) risk in women: a systematic review and meta-analysis

**DOI:** 10.1186/s12885-023-11459-0

**Published:** 2023-10-06

**Authors:** Lin Li, Baoqiang Pei, Yanyan Feng

**Affiliations:** Department of Dermatology, Chengdu Second People’s Hospital, Chengdu, 610021 Sichuan China

**Keywords:** Hormone replacement therapy, Keratinocyte carcinoma, meta-analysis, Skin neoplasms, Contraceptives, oral

## Abstract

Previous studies reported inconsistent results regarding the association between keratinocyte carcinoma (KC) and exogenous hormone therapy. This study aimed to investigate the association between the use of exogenous sex hormones and the risk of KC among women. The databases of PubMed, Ovid Medline, Cochrane, and Web of Science were searched until May 2023. A total of 5293 patients with KC and 106,424 controls were included for analysis. The meta-analysis indicated that oral contraceptives (OC) and hormonal replacement therapy (HRT) use were associated with an increased risk of squamous cell carcinoma (SCC) (OR/RR = 1.25, 95% CI 1.10 to 1.43, I^2^ = 41.6%, p = 0.080). Subgroup analysis showed that OC use increased the risk of SCC (OR/RR = 1.37, 95% CI 1.15 to 1.63), whereas no significant association was shown between HRT use and risk of SCC (OR/RR = 1.13, 95% CI 0.93 to 1.37). Additionally, OC and HRT use were linked to an increased risk of basal cell carcinoma (BCC) (OR/RR = 1.16, 95% CI 1.09 to 1.25, I^2^ = 30.1%, p = 0.188). Further subgroup analysis suggested both OC and HRT use were associated with an increased risk of BCC (OC: OR/RR = 1.13, 95% CI 1.01 to 1.25; HRT: OR/RR = 1.19, 95% CI 1.09 to 1.30). In conclusion, our findings support the hypothesis that the risk of KC among women may be affected by the use of exogenous hormones.

**Supplementary Information:**

The online version contains supplementary material available at 10.1186/s12885-023-11459-0.

## Background

Keratinocyte carcinoma (KC) is regarded as the most frequently diagnosed malignant disease among fair skin populations [1]. KC refers to the skin cancer that develops from the keratinocytes in the epidermis layer of the skin, which can be divided into two subtypes, basal cell carcinoma (BCC) and squamous cell carcinoma (SCC) [2]. BCC and SCC constitute the majority of non-melanoma skin cancer (NMSC) [3]. According to the epidemiology data, the incidence rate of KC was increasing globally, while mortality rates stable or in decline [4–6]. Tang et al. [1] reported that the incidence of KC in Ontario, Canada was 356.7 per 100,000 persons in 2017 with an increase of 30% over 14-year period. The study based on Medicare fee-for-service population in US showed that the total number of procedures for KC was 2,048,517 in 2006, while 2,321,058 in 2012, rising by 13% [7]. Kwiatkowska et al. [8] showed that the incidence of SCC was significantly increasing in England, Scotland and Northern Ireland during 2013–2018. The metastatic potential of KC is low, thus resulting in a low mortality rate, however, it is associated with high morbidity and low quality of life for patients [1, 9], and heavy health burden for many countries [10]. Increasing ultraviolet radiation (UVR) exposure dosage, as the main risk factor, contributes largely to the elevating incidence rate of KC, meanwhile KC usually occurs in the sun-exposed areas of the head and neck, followed by the trunk [11, 12].

BCC is the most common subtype of KC, accounting for almost 80% of KC [11], and the most common skin cancer among Asian and Hispanic, while second to SCC among black individuals [13]. The risk factors for BCC involve HPV (Human Papilloma Virus) infection, xeroderma pigmentosum, albinism, chemical carcinogens (arsenic and coal tar), and ionizing radiation besides UVR. Meanwhile, people with certain physical features including blond or red hair, blue or green eyes, and light skin color have a higher risk of developing BCC [14]. BCC has characteristics of low growth, locally invasion, low rates of metastases and mortality, however, the patients of BCC are at higher risk of further BCC and other UVR-related skin cancers [15]. SCC makes up 20% of KC diagnoses, with an estimation 3–16% of SCC patients developing metastasis and more than 70% of metastasis patients death [10, 11]. SCC was more common in black population, which was opposite to BCC [13]. SCC is one of the most common death causes from skin cancer only second to melanoma [16]. The disease-specific mortality of SCC ranged from 1.5 to 4% per year [17].

In addition to those risk factor mentioned, the role of hormone exposure was controversial in the development of KC. The use of exogenous hormones has been proved as a high-risk factor of breast cancer and cancers of the female reproductive tract [18, 19]. Several researchers speculated that the epidermis may become sensitive to the damage of UVR with the use of exogenous hormones therapy [20]. There were several studies on the association between KC and hormone exposure [20–26], however, results from these studies were conflicting. Based on above-mentioned condition, we conducted this systematic review and meta-analysis to investigate the association between the use of exogenous sex hormones and the risk of KC among women.

## Methods

The present study was conducted on the basis of the Preferred Reporting Items for Systematic Reviews and Meta-Analyses (PRISMA) statement [27].

### Literature search

We reviewed published articles from PubMed, Ovid Medline database, Cochrane and Web of Science. These studies were published before May, 2023. The following keywords and/or MESH terms were used: (“basal cell carcinoma” OR “squamous cell carcinoma”) AND (“oral contraceptives” OR “OC” OR “hormonal replacement therapy” OR “HRT”). Additional studies were also identified through reference lists of the retrieved articles.

### Inclusion criteria

We evaluate the article with the following selection criteria: (1) the investigator evaluated the relationship between non-melanoma skin cancer and exogenous hormones such as oral contraceptives (OC) or hormonal replacement therapy (HRT); (2) with eligible statistical parameters to estimate outcomes (odds ratio (OR), relative risk (RR)); (3) the study design was case-control or prospective cohort; (4) review papers, case reports or letters without adequate information to calculate estimated outcomes were excluded from the present study; (5) when the results reported in several models and studies presented in multiple results, we used results with higher follow-up duration.

### Data extraction and quality assessment

Data were extracted and verified independently by two researchers. The divergence was resolved by another investigator. We collected the following information from the published paper: first author and published year, type of study design, country, type of non-melanoma skin cancer, and adjusted variables. Notably, we extracted risk estimates as they were reported, either as odds ratio (OR) or relative risk (RR).

### Statistical analysis

Stata 12.0 software was employed to statistical analyses. For our meta-analysis, we primarily used the OR as the effect measure. In cases where only the RR was provided in the original studies, we treated it as an approximation of OR, particularly for events with low incidence. Accordingly, the pooled ORs (or approximated ORs from RRs) were estimated using inverse variance methods and the effects were assessed with 95% confidence intervals (CIs). What’s more, Q test and I^2^ statistic were used to evaluated heterogeneity between the enrolled researches. If the *p* value of Q test was < 0.05 or I^2^ was > 60%, we applied the random effects model for analysis; otherwise, data were pooled with fixed effects model and corresponding 95% CIs. Subgroup analysis (different study types and OC or HRT) was applied to explore the source of heterogeneity. In addition, sensitivity was imitated by removing one study at a time. The publication bias was evaluated by Begg’s test, Egger’s test and simulating the asymmetry of funnel plot.

## Results

### Included studies

**Fig. 1:**
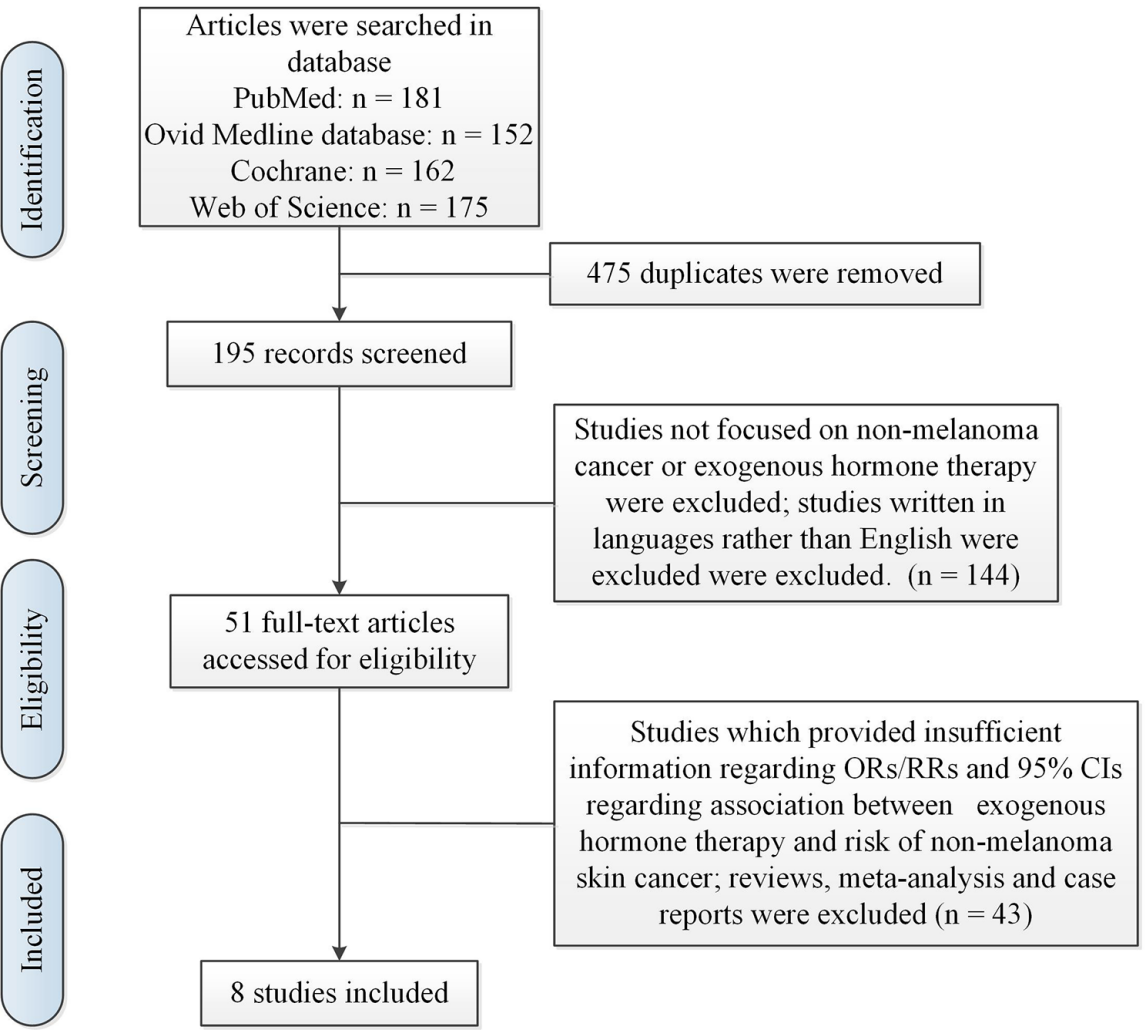
Search and selection process

Titles and abstracts were reviewed by two individuals. After removing duplicates, we found 195 English articles. After reading the abstract and titles, 144 records were excluded because of irrelevant information. Due to the research did not report the statistical parameters to estimate outcomes and other relevant information interested, 15 studies were excluded. As we failed to obtain the original data, 28 studies were discarded. Finally, 8 studies [20–26, 28] were included in present meta-analysis and they were written in English. The flow chart was displayed in Fig. 1. And the characteristics of the studies were summarized in Table 1.

**Table 1:** Characteristics of included studies

author and published year	type of study design	country	type of pathology	number of cases/ controls	mean age of cases	risk estimates (OR/RR: 95% CI)	adjusted variables
Vessey et al. (2000) [28]	cohort	UK	BCC and SCC	83/17,032	25–39	OC: Ever used: 0.9 (0.6–1.4); recently used: 0.4 (0.1–1.2); used in past: 1.0 (0.6–1.6)	age
Applebaum et al. (2009) [26]	case-control	USA	SCC	261/298	NR	OC: 1.6 (1.0, 2.5)	age, pigmentation, sunburns, sunbaths, and education
Asgari et al. (2010) [25]	case-control	USA	SCC	195/679	NR	OC: 2.0 (0.91–4.5)	known and hypothesized SCC risk factors
Langevin et al. (2011) [24]	case-control	USA	SCC	149/158	59.9 (21–80)	HRT use: Ever: 0.60 (0.31–1.06); ≤ 5 years of use: 0.69 (0.33–1.45); > 5 years of use: 0.47 (0.20–1.08); oral contraception: Ever: 1.09 (0.60–1.98); Started taking at > 22 years old: 0.75 (0.38–1.51); Started taking at ≤ 22 years old: 1.67 (0.81–3.44)	age, smoking, highest level of education, family history of cancer and number of pregnancies
Birch-Johansen et al. (2012) [23]	cohort	Denmark	BCC and SCC	1,175/76/29,875	56.3 (50–64)	HRT use: BCC: Ever users: 1.15 (1.02–1.29); Past users: 1.03 (0.87–1.22); Current users: 1.21 (1.07–1.37); SCC: Ever users: 0.96 (0.61–1.51); Past users: 0.85 (0.44–1.65); Current users: 1.02 (0.61–1.71); OC: BCC: Ever users: 1.09 (0.97–1.24); Past users: 1.09 (0.97–1.23); Current users: 1.24 (0.81–1.91); SCC: Ever users: 0.98 (0.61–1.57); Past users: 0.96 (0.59–1.54); Current users: 1.81 (0.43–7.62);	skin reaction (redness, pain, and blistering; redness, pain, and peeling; redness, then tan; or only tan), degree of freckles (none, few, moderate, or many), degree of nevi (none, few, moderate, or many), alcohol consumption (linear variable), BMI (linear variable), HRT use at baseline (never, past, or current), and duration of HRT use (linear variable)
Cahoon et al. (2015) [20]	cohort	USA	BCC	1,730/46,100	49.0 (9.3)	menopausal hormone therapy: women with natural menopause: Ever users: 1.47 (1.16 to 1.86); Past users: 1.06 (0.73 to 1.55); Current users: 1.61 (1.25 to 2.07); women with hysterectomy: Ever users: 1.12 (0.89 to 1.40); Past users: 1.25 (0.89 to 1.76); Current users: 1.10 (0.87 to 1.39); OC use: Ever users: 1.00 (0.88 to 1.13)	age, birth cohort, baseline body mass index category, alcohol consumption, MHT use, Celtic/Gaelic heritage, and lifetime average annual ambient UV radiation.
Kuklinski et al. (2016) [22]	case-control	USA	BCC and SCC	BCC: 633/550 SCC: 570/746	NR	OC: BCC: 1.4 (1.0–1.8); SCC: 1.4 (1.1–1.8); HRT: BCC: 1.0 (0.8–1.4); SCC: 1.4 (1.1–1.8)	NR
Olsen et al. (2018) [21]	cohort	Australia	BCC and SCC	BCC: 336/10,986 SCC: 85/10,986	NR	BCC: OC use: 1.06 (0.73–1.54); MHT use: 1.46 (1.07–1.97) SCC: OC use: 1.78 (0.81–3.91); MHT use: 0.79 (0.45–1.38)	Age at menarche adjusted for age at baseline, skin phototype (tanning), freckling on face at age 21, moles at age 21, skin checks by a doctor in the past 3 years and smoking status.

Abbreviations: BCC: basal cell carcinoma; BMI: body mass index; CI: confidence interval; HRT: hormonal replacement therapy; MHT: menopausal hormone therapy; NR: not reported; OC: oral contraceptive; OR: odds ratio; RR: relative risk; SCC: squamous cell carcinoma; UK: United Kingdom; USA: United States

### Association between oral contraceptive or HRT use and risk of non-melanoma skin cancer

**Fig. 2:**
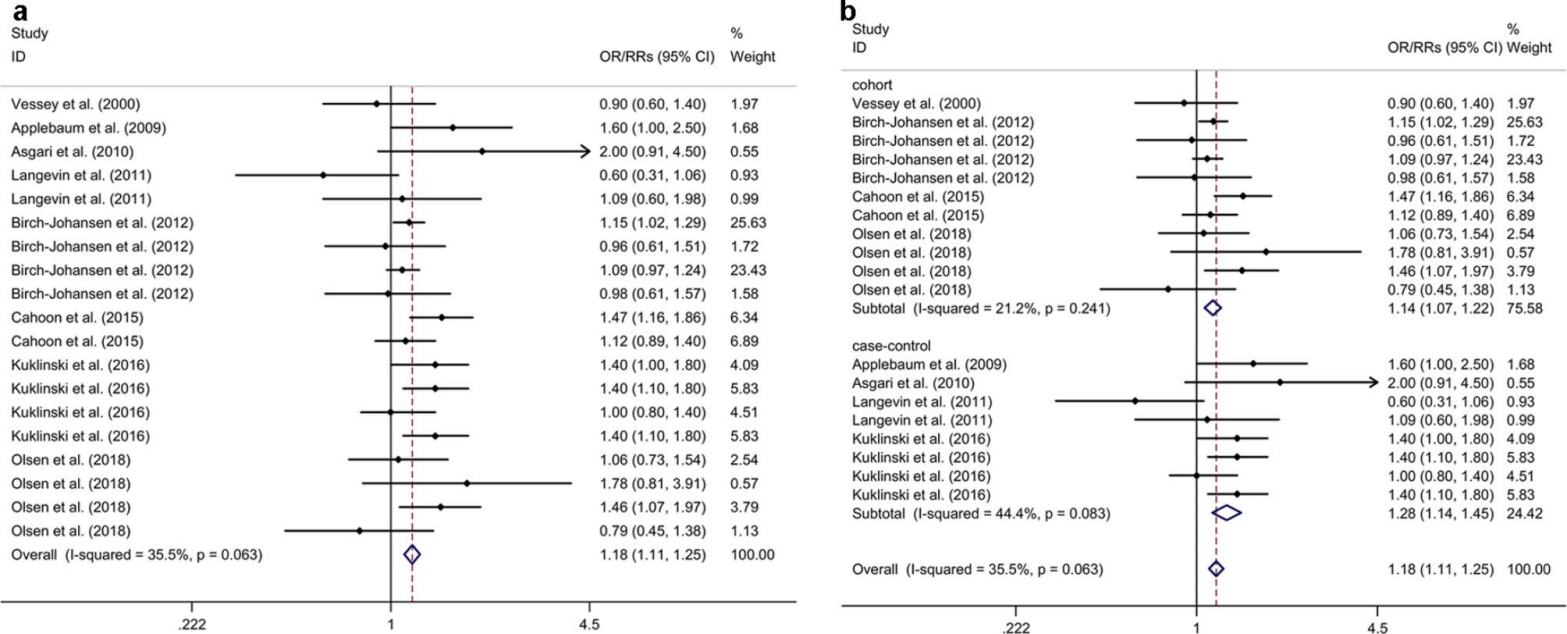
Forest plots regarding association between oral contraceptive or HRT use and risk of non-melanoma skin cancer and subgroup analysis in different study types Abbreviations: CI: confidence interval; HRT: hormonal replacement therapy; OR: odds ratio; RR: relative risk

Four cohort studies [20, 21, 23, 28] (including 3,485 non-melanoma skin cancer patients and 103,993 participants) and four case-control studies [22, 24–26] (including 1,808 non-melanoma skin cancer patients and 2,431 healthy controls (HC)) were included in the present study. The meta-analysis indicated that OC and HRT use were associated with an increased risk of non-melanoma skin cancer with a fixed effects model (OR/RR = 1.18, 95% CI 1.11 to 1.25, I^2^ = 35.5%, *p* = 0.063; Fig. 2a). Subgroup analysis indicated that OC and HRT use were associated with an increased risk of non-melanoma skin cancer in both cohort and case-control studies (cohort studies: OR = 1.14, 95% CI 1.07 to 1.22; case-control studies: RR = 1.28, 95% CI 1.14 to 1.45; Fig. 2b). Subgroup analysis indicated that both OC and HRT use were associated with an increased risk of non-melanoma skin cancer (OC: OR/RR = 1.17, 95% CI 1.07 to 1.28; HRT: OR/RR = 1.18, 95% CI 1.09 to 1.28; Supplementary Fig. 1). Sensitivity analysis indicated no change in the direction of effect while any one study was excluded from the meta-analysis (Supplementary Fig. 2). Begg’s test, Egger’s test and funnel plot showed no significant risk of publication bias (Begg’s test *p* = 0.345; Egger’s test: *p* = 0.878; Supplementary Fig. 3).

### Association between oral contraceptive or HRT use and risk of SCC

**Fig. 3:**
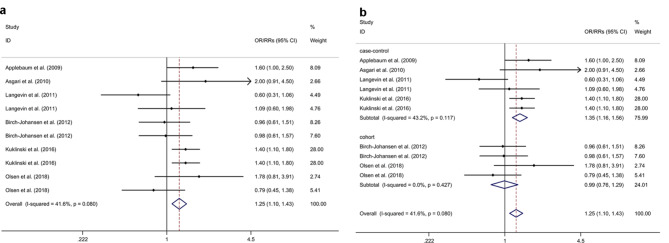
Forest plots regarding association between oral contraceptive or HRT use and risk of SCC and subgroup analysis in different study types Abbreviations: CI: confidence interval; HRT: hormonal replacement therapy; OR: odds ratio; RR: relative risk; SCC: squamous cell carcinoma

Two cohort studies [21, 23] (including 161 SCC patients and 40,861 participants) and four case-control studies [22, 24–26] (including 1,175 SCC patients and 1,881 HC) were included in the present study. The meta-analysis indicated that OC and HRT use were associated with an increased risk of SCC with a fixed effects model (OR/RR = 1.25, 95% CI 1.10 to 1.43, I^2^ = 41.6%, *p* = 0.080; Fig. 3a). Subgroup analysis indicated that OC and HRT use were associated with an increased risk of SCC in case-control studies (RR = 1.35, 95% CI 1.16 to 1.56; Fig. 3b). Subgroup analysis indicated that OC use was associated with an increased risk of SCC, whereas no significant association was showed between and HRT use and risk of SCC (OC: OR/RR = 1.37, 95% CI 1.15 to 1.63; HRT: OR/RR = 1.13, 95% CI 0.93 to 1.37; Supplementary Fig. 4). Sensitivity analysis indicated no change in the direction of effect while any one study was excluded from the meta-analysis (Supplementary Fig. 5). Begg’s test, Egger’s test and funnel plot showed no significant risk of publication bias (Begg’s test *p* = 0.929; Egger’s test: *p* = 0.336; Supplementary Fig. 6).

### Association between oral contraceptive or HRT use and risk of BCC

**Fig. 4:**
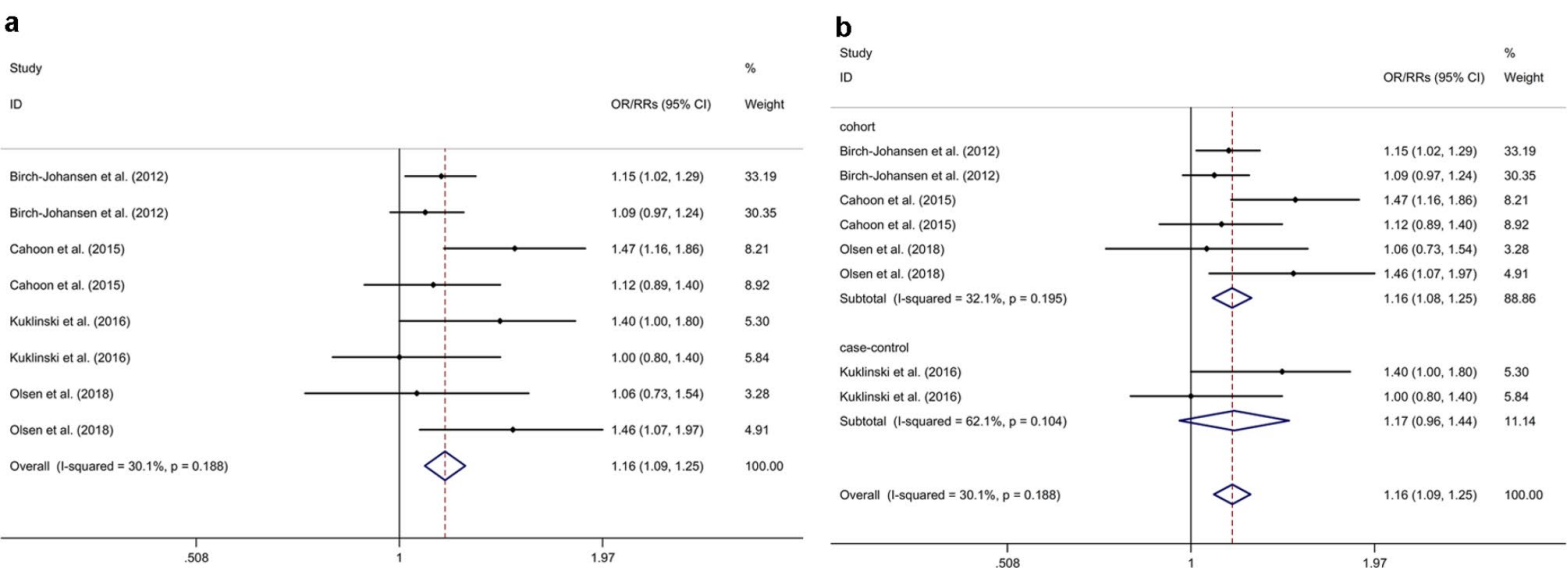
Forest plots regarding association between oral contraceptive or HRT use and risk of BCC and subgroup analysis in different study types Abbreviations: BCC: basal cell carcinoma; CI: confidence interval; HRT: hormonal replacement therapy; OR: odds ratio; RR: relative risk

Three cohort studies [20, 21, 23] (including 3,241 BCC patients and 86,961 participants) and 1 case-control study [22] (including 633 BCC patients and 550 HC) were included in the present study. The meta-analysis indicated that OC and HRT use were associated with an increased risk of BCC with a fixed effects model (OR/RR = 1.16, 95% CI 1.09 to 1.25, I^2^ = 30.1%, *p* = 0.188; Fig. 4a). Subgroup analysis indicated that OC and HRT use were associated with an increased risk of BCC in cohort studies (RR = 1.16, 95% CI 1.08 to 1.25; Fig. 4b). Subgroup analysis indicated that both OC and HRT use were associated with an increased risk of BCC (OC: OR/RR = 1.13, 95% CI 1.01 to 1.25; HRT: OR/RR = 1.19, 95% CI 1.09 to 1.30; Supplementary Fig. 7). Sensitivity analysis indicated no change in the direction of effect while any one study was excluded from the meta-analysis (Supplementary Fig. 8). Begg’s test, Egger’s test and funnel plot showed no significant risk of publication bias (Begg’s test *p* = 0.711; Egger’s test: *p* = 0.333; Supplementary Fig. 9).

## Discussion

To our knowledge, this was the first systematic review and meta-analysis to study the association between the use of exogenous hormone and KC. In this meta-analysis, exogenous hormone refers to OC and HRT. Our findings revealed that OC was associated with elevated risk of SCC, while users with HRT were prone to BCC (SCC: OC: OR/RR = 1.37, 95% CI 1.15 to 1.63; HRT: OR/RR = 1.13, 95% CI 0.93 to 1.37; BCC: OC: OR/RR = 1.13, 95% CI 1.01 to 1.25; HRT: OR/RR = 1.19, 95% CI 1.09 to 1.30). The results showed that the use of exogenous sex hormone may increase the risk of KC among females.

The potential impact of sex hormone in the development of KC may be supported by some epidemiological and laboratory studies. Estrogen receptors on the surface of keratinocytes can be activated to induce cell proliferation, further change the capacity of DNA repair [29, 30]. Cavalieri et al. [31]reported that oxidants, including DNA adducts and reactive oxygen species which were respectively produced by reactive electrophilic estrogen metabolites and estrogens, indirectly induce DNA damage, consequently resulting in the genomic and gene mutations. Furthermore, the photosensitivity reaction induced by oral contraceptive may play a potential role in the KC progression [32, 33]. The cumulative estrogen exposure may result in the phototoxic reactions in a dose-dependent manner that damage the skin cell membranes or DNA after absorbing UVR in the skin [34]. We also noticed that the women with frequently use of OC may have sexual intercourse frequently, leading to the rising risk of HPV (human papillomavirus) infection, which was associated with SCC and BCC [35]. For middle-age and old female, aging skin may be more sensitive to various harmful factors accompanying the change of hormonal status [36]. In addition, studies showed that along with social prevailing trends young girls and women enjoy the sunbathing and indoor tanning more often relative to men, even they are clearly aware of the damage from UV and use sunscreens [37, 38].

Caini et al.'s [39] study emphasized that hormonal factors do not play a significant role in the pathogenesis of NMSC among women. In contrast, our findings suggest that the use of exogenous hormones increases the risk of keratinocyte carcinoma (KC) in women. Specifically, our study identified a heightened risk of both SCC and BCC among women using oral contraceptives or hormonal replacement therapy. While both studies offer invaluable insights into the role of hormonal factors in skin cancer, our findings challenge the current understanding and underscore the need for a more nuanced evaluation, especially considering the increased prevalence and reliance on hormonal therapies among women.

In our study, we reported that sex hormones may act as a potential risk factor for KC. Given the widely use of OC globally, our findings should be verified by more powerful evidence and the impact on the risk of KC cannot be ignored. Of note, there were some limitations in our study. Fist, most of included studies were Caucasians, data from non-white women was few. Our findings may be not applicable for those women. Second, we cannot get more detail information about the use of OC and HRT, such as the first and last use, duration, type of hormones and time since drug discontinuation, etc. and aspects of patients from original literatures, such as residential history, personal sun sensitivity characteristics, body mass index, smoking habits, reproductive history, etc. These factors may influence the bias of results. Third, there were several retrospective studies included in this meta-analysis, thus recalling bias cannot be avoided. Collected information from those studies was totally based on subject recollection. The last one point worth noting, with the development of modern pharmacy, OC and/or HRT formulation that used in included studies may be different from current drug. Modern OC formulations have greatly reduced levels of estrogen [22].

## Conclusions

Our findings supported for the hypothesis that the risk of KC among women may be affected by use of exogenous hormones.

### Electronic supplementary material

Below is the link to the electronic supplementary material.

Supplementary Material 1

Supplementary Material 2

Supplementary Material 3

Supplementary Material 4

Supplementary Material 5

Supplementary Material 6

Supplementary Material 7

Supplementary Material 8

Supplementary Material 9

Supplementary Material 10

## Data Availability

The datasets used and/or analyzed during the current study are available from the corresponding author on reasonable request.
