# Supplementary material for: Exogenous hormone therapy and non-melanoma skin cancer (keratinocyte carcinoma) risk in women: a systematic review and meta-analysis
Source: BMC Cancer. 2023 Oct 6;23:946. doi: 10.1186/s12885-023-11459-0 (PMC10557205; doi:10.1186/s12885-023-11459-0)
Supplement: Supplementary file 10 — Supplementary Material 10 [file 12885_2023_11459_MOESM10_ESM.docx]

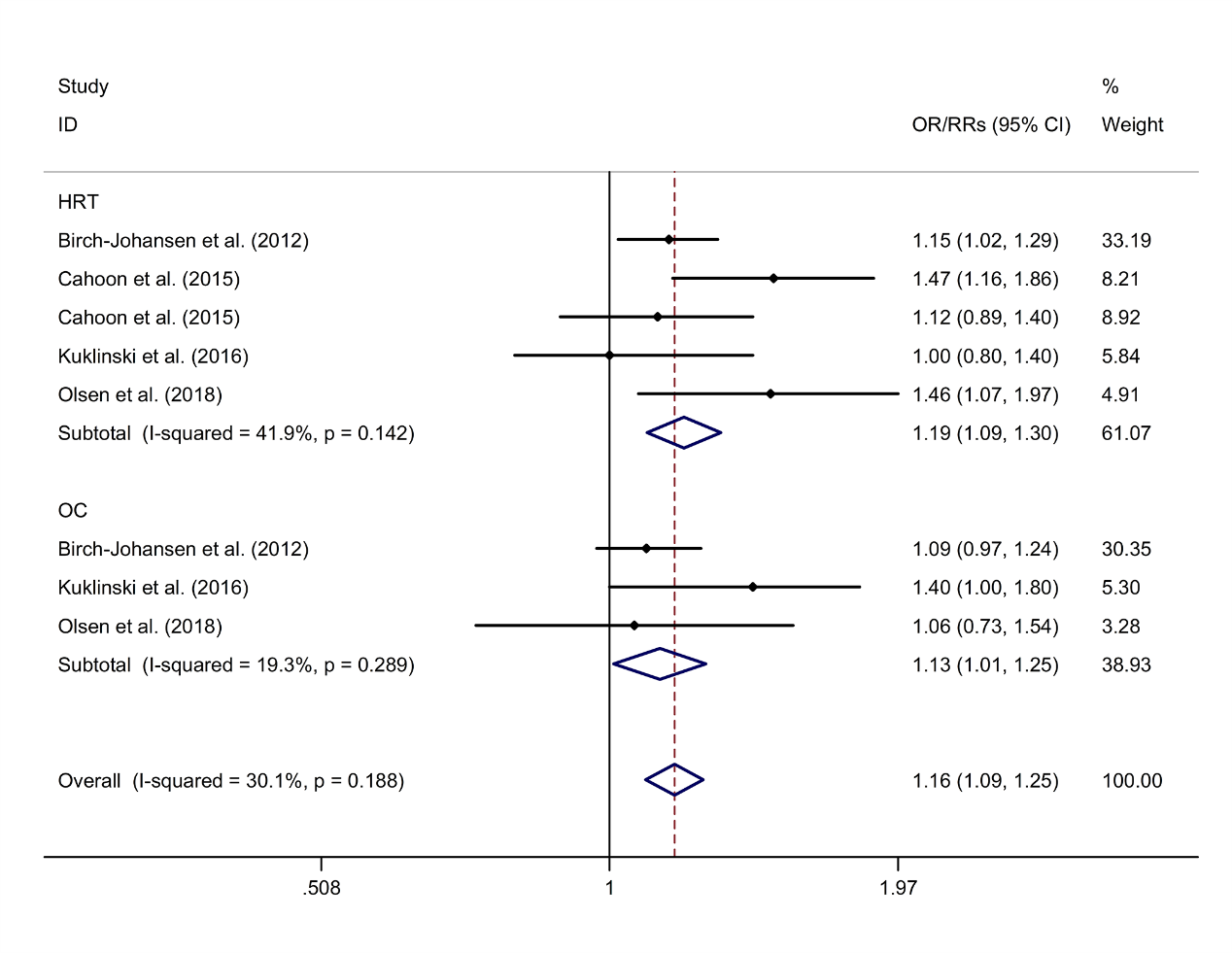


Supplementary figure 7. Subgroup analysis regarding association between oral contraceptive and HRT use and risk of BCC.
